# On Performance and Perceived Effort in Trail Runners Using Sensor Control to Generate Biosynchronous Music

**DOI:** 10.3390/s20164528

**Published:** 2020-08-13

**Authors:** Duncan Williams, Bruno Fazenda, Victoria Williamson, György Fazekas

**Affiliations:** 1 School of Computing, Science & Engineering, the University of Salford, the Crescent, Salford M5 4WT, UK; b.m.fazenda@salford.ac.uk; 2 Lucerne University of Applied Sciences and Arts, 6002 Luzern, Switzerland; musicpsychology.co.uk@gmail.com; 3 School of Electronic Engineering and Computer Science, Queen Mary University of London, London E1 4FZ, UK; g.fazekas@qmul.ac.uk

**Keywords:** algorithmic composition, biosynchronous music generation, running, physical activity, music mediated perceived effort, music perception

## Abstract

Music has been shown to be capable of improving runners’ performance in treadmill and laboratory-based experiments. This paper evaluates a generative music system, namely HEARTBEATS, designed to create biosignal synchronous music in real-time according to an individual athlete’s heartrate or cadence (steps per minute). The tempo, melody, and timbral features of the generated music are modulated according to biosensor input from each runner using a combination of PPG (Photoplethysmography) and GPS (Global Positioning System) from a wearable sensor, synchronized via Bluetooth. We compare the relative performance of athletes listening to music with heartrate and cadence synchronous tempos, across a randomized trial (N = 54) on a trail course with 76 ft of elevation. Participants were instructed to continue until their self-reported perceived effort went beyond an 18 using the Borg rating of perceived exertion. We found that cadence-synchronous music improved performance and decreased perceived effort in male runners. For female runners, cadence synchronous music improved performance but it was heartrate synchronous music which significantly reduced perceived effort and allowed them to run the longest of all groups tested. This work has implications for the future design and implementation of novel portable music systems and in music-assisted coaching.

## 1. Introduction

Trail running is an outdoor sport with a long history which has recently become the focus of academic investigation [1,2,3], particularly as the emerging field of biophilia [4] suggests that there are potentially physical and mental health benefits to engagement with the natural world. Music has been suggested as both a performance enhancer, and a mediator of psychological and physiological discomfort whilst engaging in sport [5,6]. Music can be used to increase motivation, or as a tool to help an athlete achieve a desirable mental state before partaking in sport, due to the ability of music to influence emotional states. For a full treatment of the use of music in enhancing athletic performance, the interested reader is referred to a relatively recent survey in [7]. The use of music for improving athletic performance as measured by physically quantified improved performance and mental performance improvement markers such as reduced perceived difficulty, have been the subject of a significant body of research, including findings that:

- Music can mediate physical responses to pain [8].
- Musical cues, particularly the sonification (Sonification is a practice of auditory display whereby data are auralized. A simple example would be an alarm) of biomedical data, can encourage good form in strength and conditioning activities [9].
- Music can improve athletic performance, and reduce the perceived effort involved [10,11].
- Appropriate music selection is not trivial [12,13].

Previous work has evaluated the role that music selection might play in mediating heartrate and perceived exertion in running for 20 min periods [11], in a treadmill condition [14], and in more recent studies investigating affectively-driven music selection on the basis of biosensing [15], whereby music selection is made according to a target heartrate. Music selection has been shown to enhance running performance, by encouraging optimal running cadence by means of music [16]. The selection of music is critical to informing the research documented in this paper. Systems for automatic music selection and curation now exist [17], but our particular focus is on harnessing the power of algorithmic music generation [18], which provides techniques that can power systems to create new music with no predetermined time-frame, that might operate synchronously with an athlete’s own biosignals. When listening to our favourite music, our bodies respond physically, inducing reactions such as pupil dilation, increased heart-rate, blood pressure, and skin conductivity [19,20]. These biomarkers are particularly interesting as existing work has shown that there is a neurological and physiological connection between emotional state, human performance, and music listening [21], which recent advances in wearable sensor technology and portable programmable music systems might now potentially address. A number of suggestions for optimal running cadence have been made (where cadence, or gait, is the number of full cycles per minute, i.e., by both feet) but these values are highly variable, dependent on individual stride length, technique, and other bio-mechanical factors [22]. Nevertheless, cadence selection has been shown to reduce running-related injuries and reduction of time taken to fatigue [23,24]. Treadmill based experiments do not necessarily translate well to real-world running conditions outdoors, and as such we will focus on the design of an experiment considering the use of music in an ecological context-outdoor running. The prototype system we have designed, described in Section 2, is the subject of an experimental analysis in this paper. We believe that, in future, these types of system also have the potential to be adapted to real-time biosynchronous feedback (for example, audio-based coaching), a use case which music and trail running are particularly well suited to from a user safety perspective because of the lack of vehicle traffic or the need for visual interfacing.

### 1.1. Synchronous and Asynchronous Music as Affective Correlates and Athletic Performance Enhancers

Specific choices of asynchronous classical music have been shown to reduce the heart rate and perceived exertion (amongst other physiological measures) of athlete’s during running [25], wherein participants exhibited decreased heart rate and blood pressure in the music condition. Some more recent work suggests that asynchronous music was less motivating than synchronous music for treadmill running [26]. Beyond running specific evaluations, asynchronous music was suggested to have a greater degree of influence on valence (valence is often used as a means of quantifying the positivity of a particular emotional state) [27,28]. Some research suggests that synchronous music has a greater influence on mood [29], promotes greater endurance [30], and may further reduce limbed discomfort and increase arousal level (arousal as an affective correlate is a means of quantifying the activation strength of a particular emotional state) [31]. There is also increasing evidence of a correlation between athletic endurance and levels of valence [32].

In the existing literature, three specific functions of music have been widely reported as having a significant influence on athletic performance:

- Auditory–motor entrainment, i.e., the tendency of the listener to synchronise their own movements with musical features.
- Dissociation: the ability to distract an athlete from discomfort by means of listening to music.
- The ability of music to increase emotional arousal (where arousal is synonymous with emotional intensity or activation strength)

Music has been shown to be able to narrow an athlete’s focus and attention whilst exercising, diverting them from unpleasant sensations such as fatigue [31,33], and from other distractions like environmental cues (for example in the case of trail running, bad weather might be of particular concern as opposed to treadmill or laboratory based experiments). There is a psychological parallel with the sensation of total immersion or flow [34], wherein the athlete is so involved in the activity, i.e., listening or exercise, that other such distractions are pushed out of their cognitive processing. These distractions might include, in the case of trail running, thoughts about competitors, or a focus on the runner’s own form and technique, which would ideally become autonomic processes. Music can thus be used to reinforce skill development in the athlete [5]. However, there could be cases where this development might be hindered by music, for example when music becomes a distraction in its own right. Again, such findings reinforce the case for careful curation of music selections to maximize athletic performance.

A range of musical features have been proposed as correlates for emotional and perceptual states in listeners, including rhythmic responses whilst exercising [31]. Perhaps unsurprisingly, musical rhythm has been found to be influential in many cases, both by encouraging motor responses which are synchronous with the temporal characteristics of music (such as when people dance to music), and also with asynchronous effects [27]. Tempo, for example, can be used synchronously by matching the speed of a piece of music to an athlete’s heartrate [35,36], or asynchronously by switching between slow and fast tempo in order to try and heighten the athlete’s motivation and increase work output, with marked effects shown across a range of sports [25,26,27], including rowing [37] volleyball [38] and cycling [39].

Musical features which have a strong correlation with induced or perceived arousal include tempo (higher tempi are often correlated with higher arousal), mode (where minor keys are often associated with lower valence, and major keys with higher valence, or positive states), and rhythmic density (with less dense patterns being associated with decreased arousal) [40]. Sporting activities, particularly those with an element of competition, are very likely to cause an increase in arousal for participants and spectators alike. Music can be used to stimulate or attenuate arousal (e.g., to calm an athlete down before undertaking competitive activity), or to increase arousal before or during the activity itself [41]. This can be imagined as a Yerkes-Dodson curve wherein stimulating or attenuating effects of music operate on an inverted ‘U’ shape in relation to performance, rather than as a linear function. Stimulation/music induced arousal can be a positive effect in relation to performance, but only up to a point. Note, this is not the same as saying music can become a distraction, this is a separable attention-related effect. In brief terms, when performance is suboptimal it is possible that music may have led to over or under stimulation. Musical amplitude and tempo have both been previously evaluated in the context of improvement in running performance [10], whereby louder, faster music resulted in a quicker running pace. Previous research investigating the effect of synchronous music on movement found a marked ergogenic influence on running shorter distances [42], with a significant increase in work output (faster sprint times) and endurance capacity [31]. Whilst we do not draw any specific hypothesis from the literature regarding the influence of music on running performance across gender, we designed our experiment to also capture this information and to examine whether there were any significant gender differences in our participants. Specifically, we examine gender differences in a range of synchronous and asynchronous music conditions as part of our experimental analysis in Section 3.

### 1.2. Overview of Algorithmic Composition

A huge variety of music generation systems exist, with perhaps the earliest example being Mozart’s Musikalisches Würfelspiel, the ‘dice game’, which uses the roll of a dice as a control signal to inform the selection of pre-composed musical segments. Music generation by algorithmic means is not novel. Algorithmic composition systems include transformative systems, wherein source musical passages are adjusted according to various functions (transposition, retrograde inversions, etc.), and purely generative systems, whereby rulesets and constraints are used to modulate otherwise randomly generated source material. The latter approach is particularly well suited to data-driven approaches. For the purposes of the work described here, the use of algorithmic composition over selection of existing musical stimuli has the advantage over ‘traditional’ music selection in that we can create continuous music playback (hence no pauses between song selections whilst our participants are running), and most crucially the ability to be precisely synchronous with an individual’s given heartrate or running cadence as measured using a lightweight wearable biosensor. Our work is closest in this regard to recent work using biosensors to control music based on emotional estimation [43], and the interested reader might want also see related evaluation of sensor type and mappings with specific musical examples (singing) [44], or in a broader range of work in the recent review of musical interfaces, which includes biosensor interfaces, given by Frid [45].

## 2. Materials and Methods

The system described and evaluated here, namely HEARTBEATS, was developed to track a runner’s cadence and/or heartbeat to generate synchronous music which might promote optimal running performance in the listener. It makes use of generative music production techniques, as described in [46], to create new music according to a second order Markov model specifying rhythmic and melodic musical feature generation in real-time with varying degrees of repetition. This is of note because previous work has noted how repetition can create a range of emotional responses in listeners [47]. We are particularly interested in the use of music with athletes in an ecological context (i.e., real-world outdoor environment), and the potential of biophysiologically synchronous, computer-generated music to aid performance. Cadence had been documented in the literature as a prominent feature, but matching optimal cadence to running (between 150–180 steps per minute) which the reader should note would be further challenging in a system using traditional music selection. There are simply not vast quantities of existing music at 180 bpm. In HEARTBEATS, music is generated at a synchronous pace for the athlete based on either their heartrate or cadence whilst running. Latency is minimal but transformations occur at bar breaks, which gives the impression of being instantaneous at all but very slow tempo values. We therefore designed a system to investigate the relative performance between heartrate and cadence synchronous music by experiment with the following research question: Does cadence or heartrate synchronous algorithmically generated music improve athletic performance or reduce perceived exertion in trail runners?

### 2.1. Music Generator

**Figure 1:**
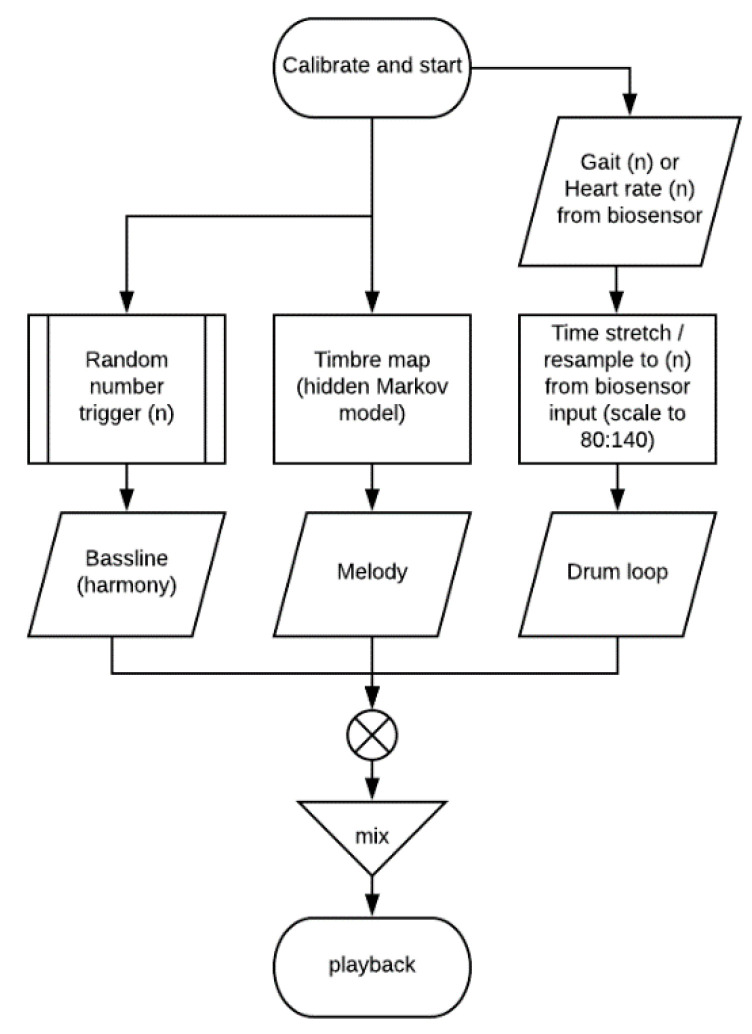
Overview of music generation system. Tempo, melody, and timbral features in the generated music output are modulated according to biosensor input from individual users. Timbre mapping is by 2nd order hidden markov model.

An overview of the music generation system is shown in Figure 1.

The rhythmic, melodic, and timbral features of the generated music are modulated according to a combination of biosensor input for tempo and metrical data, and random number selection of a series of prescribed musical feature mappings based on the features described above. A preloaded selection of source sound files is combined and mixed in various combinations, and can also be resampled, time-stretched, or pitch shifted, according to the desired cadence or heartrate. These effects have a result in the perception of the music speeding up or slowing down, depending on the desired tempo.

### 2.2. Experiment Design

A group of 57 volunteers were recruited from a local (county level) trail running club to participate in an experimental trial of the system on a real-world trail course. These volunteers were recruited via the club social secretary as part of a series of social runs-volunteers were not screened according to ability or experience. The music condition was assigned randomly. Of the 57 trials returned, 29 were carried out with cadence synchronous music, and 28 with heartrate synchronous music, however not all of this data were used in the final analysis (54 out of 57 participants data were used; for further detail, see Section 3).

**Figure 2:**
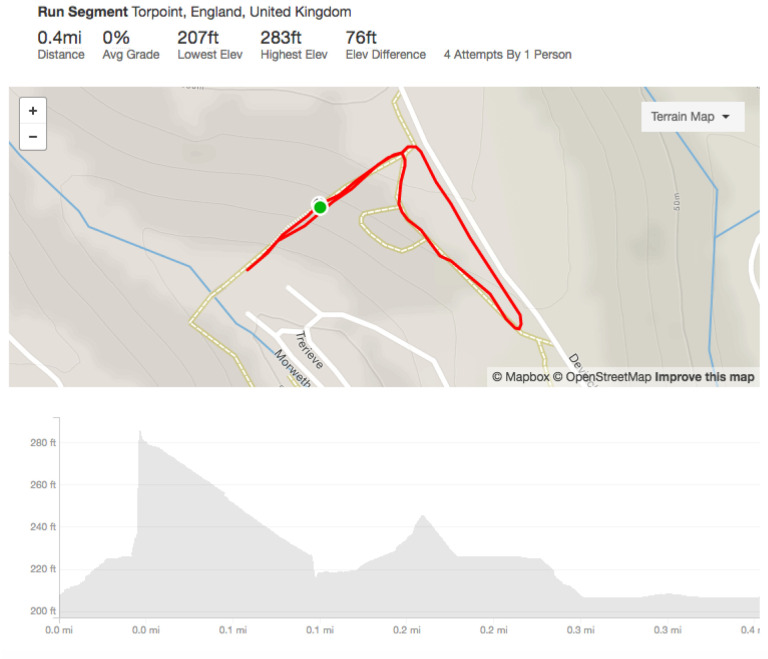
Course and elevation profile—note extremely steep grade in sections.

Runners were self-timed on a course segment with 76 ft of elevation including a steep section with a 26% grade, as shown in Figure 2. Participants were instructed to continue until self-reported perceived effort went beyond an 18 on the Borg scale from 6–20, with 6 being no exertion at all, and 20 corresponding to maximal exertion. Participants were given a short training session giving instruction on the use of the scale prior to beginning the experiment. The course segment was chosen to be representative of a variety of trail race conditions, including fell and mountain racing, and trials were conducted over a weeklong period in December 2017, with runners submitting their results via a commonly used social networking tool. As such, there is an element of trust involved in any individual reporting their own time and distance honestly, with the expectation being that this was an experiment, not a race, and runners should take part within the framework of an ‘honour’ system.

The study design was granted ethical approval by the Physical Sciences committee of the host institution at the time of conducting this experiment. Participants all provided informed consent and data were stored with compliance to GDPR regulations, particularly the anonymization of biophysiological measurement data.

### 2.3. Sensor Type and Data

The heartrate sensor used was a multi-wavelength photoplethysmograph (PPG) by Osram (the SFH-7060). This provides heart rate and pulse oximetry using a combination of green, red, and infrared light, digitized at 24-Bit to a single channel before being high pass filtered. Distance, cadence, and pace information are determined by combination of timecode, accelerometer, motion sensor/gyroscope to estimate step count, and GPS information. Sensor data were synchronized via Bluetooth.

## 3. Results

Inspection of the data revealed three outliers in the female dataset, all using cadence synchronous music. Their data were found to be more than 1.5 times the interquartile range below the lower quartile. Data collected from individual runners were anonymised so examining other factors (e.g., runners’ previous history, etc.) was not possible, but the three runs in question took place on 20 December 2017. Not all the runs took part on the same date. The historic weather report for the region on this date showed a higher than average west–south-westerly wind (>10 mph) and thunderstorms in the area. This might have contributed to the duration and subsequent distance in this group of data, especially as parts of the course segment are at a significant elevation compared to the surrounding area, and therefore are relatively exposed to wind. Equally, these participants may not have enjoyed the generated music-the current system makes no attempt to accommodate individual musical preferences.

For the remaining analysis, data counts were as follows: heartrate synchronous condition runners: 15 females and 13 males; cadence synchronous condition runners: 13 females and 14 males. Analyses of variance tests have thus used unbalanced designs.

Analysis of data were split into pace per mile, duration of the run, distance covered and average runner heartrate. The analysis considered main and interaction effects for the tracker type and runner gender.

Unless otherwise indicated, the homoscedacity and normality of residues assumptions for the analyses of variance were tested with Levene’s test for homogeneity of variance and Shapiro–Wilk normality test, respectively.

### 3.1. Analysis for Pace Per Mile

**Figure 3:**
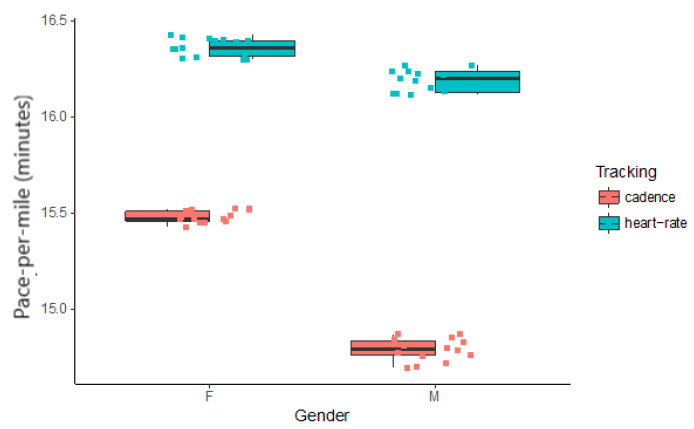
Boxplot for Pace per Mile (minutes per mile) indicated by Gender and Tracking type.

Pace per mile data (Figure 3) were tested and found to have homogenous variance (df = 3; F = 2.6189; p = 0.06079) and a normal distribution of residues (W = 0.95741; p = 0.04932). A type III two-way unbalanced design ANOVA was modelled (Table 1) with pace per mile as the dependent variable and Gender and Tracking as independent variables. Both interaction and main effects were found to be highly significant. Effects sizes (indicated in Table 1 as η^2^) were large for gender (η^2^ = 0.148) and tracking (η^2^ = 0.805) and small for their interaction (η^2^ = 0.041).

Smaller values indicate faster running pace. Clearly, cadence synchronous music affords faster running pace than heartrate synchronous music, for both genders. The differences between genders are more marked when using cadence synchronous music. To establish whether there is a significant difference between the heartrate results across gender, a *t*-test was calculated. The heartrate data was found to be normal for each of the samples using a Shapiro–Wilk test (W = 0.89689, p = 0.08532 for the Female group; W = 0.88226, p = 0.07653 for the Male group). Equal variances were found across the samples with an F-test for the equality of variances (F = 0.64684, num df = 14, denom df = 12, p = 0.4331). A significant difference in pace across gender for the heartrate synchronous condition was found (t = 8.6956, df = 26, p = 3.577 × 10^−9^).

### 3.2. Analysis for Duration of Run

**Figure 4:**
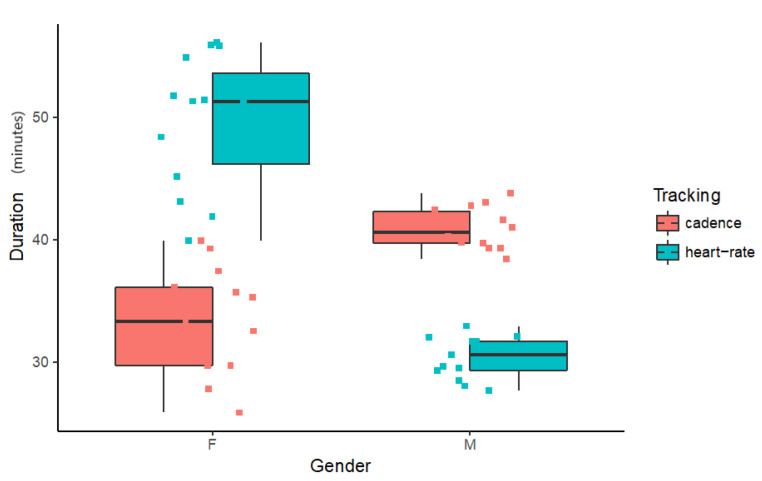
Boxplot for Duration in minutes indicated by Gender and Tracking type.

Duration of run data was tested and found to violate the assumption for homogenous variance (df = 3; F = 5.2405; p = 0.00314). This can be seen in Figure 4 which shows that data for males have a smaller variance than that for females.

Given the necessary assumption for a two-way ANOVA is violated, the data were tested for each gender separately.

#### 3.2.1. Duration of Run for Females

A Levene’s test for equality of variance indicates this assumption is met for the Female data (df = 1; F = 0.2297; p = 0.6358) and the assumption for normal distribution of residues (W = 0.94352, p = 0.1358) is also met. A type III unbalanced ANOVA was modelled (Table 2) with duration as the dependent variable and tracking as independent variable for the female data. Tracking was found to be highly significant with a large effect size (η^2^ = 0.75). Females ran significantly longer for the heartrate tracking condition.

#### 3.2.2. Duration of Run for Males

Equal variance (df = 1; F = 0.0621; p = 0.8052) and normal distribution of residues (W = 0.9493, p = 0.2062) were found for the Male duration of run data. A type III unbalanced ANOVA was modelled (Table 3) with duration as the dependent variable and tracking as independent variable for the male data. Tracking was found to be highly significant with a large effect size (η^2^ = 0.914). Males ran significantly longer for the cadence tracking condition.

#### 3.2.3. Duration of Run-Females vs. Males

As seen before, the variances in the data across gender are not homogeneous. A Wilcoxon rank sum test (W = 196, p < 0.001) indicates that females using the heartrate tracker ran significantly longer than males using the cadence tracker (see Figure 4).

### 3.3. Analysis for Running Distance

We considered data for running distance solely for male participants. This is due to 14 Females having all completed exactly 2.84 miles in the heart rate condition, presenting a data subset with extremely small variance. It is difficult to explain this without considering human factors. Trail running as an exercise is both communal and competitive, and as such it could be that this group were either running together, or racing each other (or potentially even racing in ‘packs’). Therefore, it could be that these participants may not have reached their individual maxima, instead stopping when their peers did. This type of free-choice qualitative data were not collected from the participants, and might reasonably be a useful recommendation for others wishing to conduct such experimental work outside of the treadmill or the laboratory. However, assuming one of the latter conditions occurred, we focus the remaining analysis on the Male participants.

**Figure 5:**
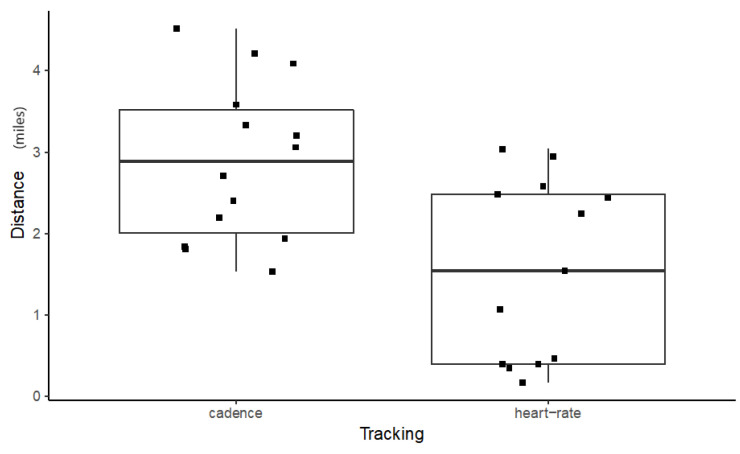
Boxplot for Distance completed (in miles) for Males by Tracking type.

Male distance data were analysed for equal variance and normality. Although equal variances were found (df = 1; F = 0.9354; p = 0.3427), the distribution of residues was not normal (W = 0.90741, p = 0.01984). Differences between tracking conditions were tested for using a two-sided Wilcoxon rank sum test (W = 144, p = 0.0101) which indicates a significant difference between heartrate and cadence tracking for distance completed. Males using the cadence tracking ran for significantly longer than those using the heartrate tracker (Figure 5).

### 3.4. Analysis for Heart Rate

**Figure 6:**
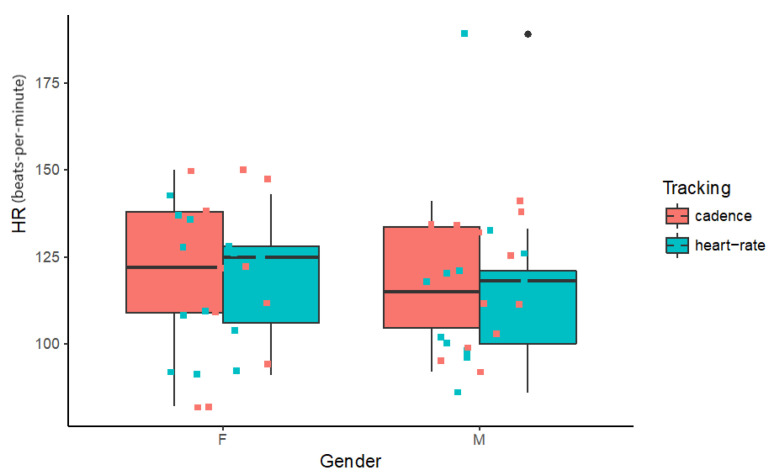
Boxplot for individual average heart rate (bpm) by Tracking type across Gender.

Average heart rate data collected for each individual were analysed for each tracking condition across gender. This data subset was found to have homogenous variance (df = 3; F = 0.3071; p = 0.8201) and a normal distribution of residues (W = 0.96033; p = 0.06691). A type III two-way unbalanced design ANOVA was modelled (Table 4) with heart rate as the dependent variable and gender and tracking as independent variables. No interaction or main effects were found to be significant. Given the low power of this analysis (Table 4, last column), it is unknown whether we might incur a type II error in stating that no differences in heart rate exist between genders and tracking type under the experimental conditions tested. Average heart rate across all conditions is 117 bpm. Figure 6 shows these data.

## 4. Discussion

A large body of work documenting improved athletic performance in response to music exists. Previous work specifically documenting running has included synchronous and asynchronous music. For the work presented here, the growing field of affectively driven algorithmic composition has been adapted to the task of musical stimulus generation to create biophysiologically synchronous music, specifically heartrate and cadence, in order to evaluate the effect of such a system on perceived effort and work output in a specific ecological context: outdoor trail running.

A computer system for generating a continuous stream of music with a varying tempo was designed and adapted to a control signal input derived from a combination of biophysiological readings measured using wearable biosensors. Specifically, PPG sensor data in combination with gyroscope and GPS data allowed for heartrate, pace, and cadence data to be estimated and monitored in real-time, and used as the control signal for the tempo parameter of the algorithmic music generator. Previous research effort has considered the positive effect of music on athletic performance. However, recent advances in wearable sensor technology and portable music generation tools provide the opportunity to customise a musical soundtrack to an individual’s own biomarkers. This paper documents one such attempt, with a real-world experiment evaluating the system in use by a group of trail runners.

Our experimental results suggest that the optimization of synchronous music for trail running is likely to depend on the goals of the runner (whether they want to improve pace, distance, or duration) and their gender.

For pace, cadence synchronous music seems to afford better performance than heartrate synchronous music regardless of gender, and this improvement is more marked for males. For both types of tracking, males register significantly faster paces than females, but males using heartrate tracking ran slower than females using cadence tracking. For duration, females ran for longer when using heartrate synchronous music, whereas males ran for longer when using cadence synchronous music. Notably, females using heartrate synchronous music ran for longer, on average, than any of the other groups, including males using their best performing cadence synchronous music. It might be that the Females taking part treated the exercise as a team effort, running in a “pack”, whilst the Males treated it more as a race-work, suggesting there are gender differences in competitive orientations for running [48]. The interaction between different conditions across gender is curious, and methodologically, it would have been useful to conduct a qualitative set of interviews with participants afterwards to help shed further light on these results. There is literature which explores gender differences in runners [49], but not with regards to our specific biosensor music cases, and as such we can only speculate as to the underlying reasons. One possibility, noted in literature, is that Females may be more susceptible to involuntary musical imagery, so-called “earworms” [50], which can be perceived positively or negatively: it might be that the heart-rate synchronous condition caused more earworm type responses in some of the Female participants, though this is purely speculation on our part. Previous research shows that people of any gender react in a similar way in terms of arousal when reporting earworms in real music [51]. Usable data for distance was available for the male group only, showing that cadence synchronous music affords significantly longer effort before exertion.

In summary, cadence synchronous music improved overall running performance for male subjects, who ran significantly faster, further and for longer than any of the other groups, except the female group using heartrate synchronous music who ran for significantly longer than anyone else.

Data variance in the run duration was found to be much wider for females than for males (whereas this was not the case for pace data), perhaps suggesting that, under our experimental conditions, the relative ranges of endurance are less varied for the male participants than for the female ones. We found no significant differences between gender or tracking type in the average heartrates measured for each individual, suggesting that the benefits afforded might be independent of the level of effort of the individual.

The results presented here are comparable with other research that has noted lower perceived exertion when (male only) runners use synchronous music [52], lower heart rate, and increased distance in (male and female) runners using synchronous but not asynchronous music [26], and in the use of musical tempo to influence running cadence (with specific marked gender interactions) [53]. Such findings are closer in the spirit of the activity to the trial conducted here than other work looking at, for example, sprinter performance on a track [42] though similar interactions with synchronous music conditions were also reported.

This study only investigated one iteration of the music generator, but a number of other musical features could be incorporated in a more advanced system. For example, taking into account individual musical preferences using the Short Test of Musical Preferences questionnaire [54] prior to participation, would allow training the generator on an individual’s own selection of musical material and testing whether improved performance is associated with musical preference. This is not trivial and remains a significant topic of research, particularly when considering how the emotional state requirements of an individual may change given the timing of their session, e.g., even at a simple level this would necessitate changes between an emphasis on motivation in the pre-exercise state, or recovery post-exercise. Moreover, due to the wide range of tempi required by the generator in this trial (typically varying from 86–150 bpm), it is likely that this system would not be appropriate for certain activities, for example, highly anaerobic activity with heart rates that would be beyond tempo ranges that might typically be considered musical. These challenges might be solved by considering different metric levels in the generation of musical material, e.g., the use of 12/8 time, so that strong beats do not have to fall solely on the first beat of the bar, allowing for divisions of the target cadence.

Such solutions remain the subject of further work focusing on the design of the music generator, rather than the underlying possibilities afforded by the use of the biosignal and resulting performance feedback loop. Specifically, whilst generative music technology has the potential to produce infinite soundtracks in sympathy with an athlete’s biosignals, this need not be restricted to the extracted biosignal value of the athlete. Thus, in future, trials with target tempi could be conducted, i.e., encouraging the runner to move at a specific goal speed, rather than following the runner’s speed. For example, to generate music with optimal tempo as a function of current heartrate or cadence, and optimal heartrate or cadence, depending on the stage of training (warm-up, main training, cool-down, or particular heartrate target zones depending on the athlete’s goal). This suggests the very real possibility of ‘smart music coaching’ systems, according to heartrate (for example, in maximal fat burning) or cadence (in injury treatment or seeking maximal athletic performance).

## Figures and Tables

**Table 1:** Type III 2-way unbalanced design ANOVA for Pace per Mile.

	Sum Sq	Df	F Value	Pr(>F)	Eta_sq	Power
**(Intercept)**	3115.20	1	1,267,327.46	<2.2 × 10^−16^		1
**Gender**	3.20	1	1300.20	<2.2 × 10^−16^	0.148	1
**Tracking**	5.44	1	2214.04	<2.2 × 10^−16^	0.805	1
**Gender: Tracking**	0.92	1	372.29	<2.2 × 10^−16^	0.041	1
**Residuals**	0.13	51				

**Table 2:** Type III ANOVA for Duration of Run for Females.

	Sum Sq	Mean Sq	Df	F Value	Pr(>F)	Eta_sq	Power
**Tracking**	1844.1	1844.1	1	77.84	<2.69 × 10^−9^	0.75	1
**Residuals**	615.9	23.7	26				

**Table 3:** Type III unbalanced design ANOVA for Duration of Run for Males.

	Sum Sq	Mean Sq	Df	F Value	Pr(>F)	Eta_sq	Power
**Tracking**	749.2	749.2	1	265.3	8.06 × 10^−15^	0.914	1
**Residuals**	70.6	2.8	25				

**Table 4:** Type III 2-way unbalanced design ANOVA for individual average heart rate.

	Sum Sq	Df	F Value	Pr(>F)	Eta_sq	Power
**(Intercept)**	188,884	1	425.9797	<2.2 × 10^−16^		1
**Gender**	68	1	0.1539	0.6965	0.002	0.06
**Tracking**	97	1	0.2195	0.6414	0.003	0.07
**Gender: Tracking**	26	1	0.0591	0.8089	0.001	0.057
**Residuals**	22614	51				
